# Caregiver and Pediatric Clinician Perspectives on Artificial Intelligence for Language Services

**DOI:** 10.1016/j.acap.2025.102887

**Published:** 2025-07-05

**Authors:** Gabriella Ficerai-Garland, Peyton Groves, Elena A. Puccio, Sabrina Bruno, Henry Hoffman, Nadia Pineda Villegas, María Cecilia Q. Dancisin, Alisa Khan, K. Casey Lion, Diego Chaves-Gnecco, Mona Diab, Maya I. Ragavan

**Affiliations:** University of Pittsburgh School of Medicine, Pittsburgh, Pa; University of Pittsburgh School of Medicine, Pittsburgh, Pa; University of Pittsburgh School of Medicine, Pittsburgh, Pa; University of Pittsburgh School of Medicine, Pittsburgh, Pa; University of Pittsburgh School of Medicine, Pittsburgh, Pa; Division of General Academic Pediatrics, University of Pittsburgh School of Medicine, Pittsburgh, Pa; UPMC Children’s Hospital of Pittsburgh, Pittsburgh, Pa; Division of General Pediatrics, Boston Children’s Hospital, Boston, Mass; Department of Pediatrics, Harvard Medical School, Boston, Mass; Department of Pediatrics, University of Washington School of Medicine, Seattle, Wash; Center for Child Health, Behavior and Development, Seattle Children’s Research Institute, Seattle, Wash; Division of General Academic Pediatrics, University of Pittsburgh School of Medicine, Pittsburgh, Pa; Language Technologies Institute, Carnegie Mellon University, Pittsburgh, Pa; Division of General Academic Pediatrics, University of Pittsburgh School of Medicine, Pittsburgh, Pa

**Keywords:** artificial intelligence, health inequities, language equity, languages other than English

## Abstract

**Objective:**

Children and caregivers who use languages other than English (LOE) for pediatric health care experience inequities and poorer overall health. Advancement of large language models has raised questions about the future use of artificial intelligence (AI) for language access. This study explored the perspectives of caregivers who use LOE and pediatric clinicians on using AI to address unmet needs in health care translation and interpretation.

**Methods:**

We conducted semistructured individual interviews with caregivers of pediatric patients who use LOE and pediatric clinicians about the use of AI language technologies in health care. A phone interpreter or fluent team member was used for LOE interviews. Recordings were transcribed, translated as needed, coded, and analyzed using inductive thematic analysis.

**Results:**

Twenty caregivers using 11 different LOE and 22 clinicians participated. Themes were consistent across participants, though clinicians were more familiar with the concept of AI. Participants reported use of technologies (eg, Google Translate) for written communication and situations where verbal interpretation was perceived to be inadequate. They were concerned about AI accuracy, privacy, and loss of empathy, but hoped it could provide real-time document translation and more convenient verbal communication. Ease of use, validation, and equitable creation and access were critical for use.

**CONCLUSIONS:**

Caregivers and clinicians were open to using AI to fill gaps in translation and interpretation; however, robust validation of AI technology and complementary use with human interpreters and translators is needed. Future research, practice, and policy should focus on integrating AI while investing in human translation and interpretation.

People with limited English proficiency (LEP), defined as those who report speaking or understanding English less than very well on a US census question,^1^ experience health care inequities due to interpersonal and structural discrimination.^2,3^ Children ages 5 to 17 make up 9% of the LEP population,^4^ and nearly 6 in 10 children of immigrants have a caregiver who identifies as having LEP.^5^ In the health care setting (and for this study), we use the term languages other than English (LOE) rather than LEP to describe caregivers who use another language in health care contexts in an effort to use more inclusive, strengths-based language, and to more accurately describe the population, which is not identified using a US census question.^6^ In addition to inadequate access to care, patients and families who use LOE are more likely to experience communication–related medical errors and serious adverse events, resulting in increased morbidity and mortality.^7–9^

Provision of linguistically equitable services—including interpretation (spoken/signed) and translation (written)—is an evidence-based solution to dismantle health inequities experienced by caregivers who use LOE and their children.^10,11^ Human interpreters and translators are the mainstay of cross-language communication^12^; however, gaps in translation and interpretation exist.^13^ While prior studies have shown that popular language technologies such as Google Translate are not acceptable for use in health care settings due to limited accuracy,^14,15^ development of large language models such as ChatGPT^16^ has called into question the potential to use artificial intelligence (AI) for communication in health care. Recent studies evaluating ChatGPT have found it to be flexible and adaptable, allowing it to reorganize text to generate translations that accurately capture original meaning and tone.^17–19^ However, concerns remain regarding variability in accuracy and capabilities across languages.^18–20^

As AI language technologies evolve, it is important to consider the perspectives of caregivers and clinicians who may use these technologies now and in the future. Prior studies have explored clinician and patient attitudes toward communication technology^21^ and AI in health care,^22^ but studies of caregiver and clinician attitudes toward AI for language services in pediatric health care settings are limited. The goal of this qualitative study was to explore perspectives of caregivers who use LOE and pediatric clinicians around unmet language access needs, AI’s current use, strengths, and weaknesses, and recommendations for future implementation.

## Methods

We conducted virtual, semistructured, individual interviews with caregivers who use LOE and pediatric clinicians. For this study, we defined AI as any technology that can perform tasks that normally require human intelligence, including Google Translate, ChatGPT, or institutional Health Insurance Portability and Accountability Act–protected AI software. We focused on Google Translate given widespread familiarity across caregivers and clinicians, and ChatGPT given its ability to tailor responses based on context and instructions and to interpret and generate text, sound, and images.^23^ We used a descriptive qualitative approach. Descriptive qualitative research is useful for hypothesis generation, particularly in the context of a new phenomenon such as AI technologies. It is also useful to center participants’ experiences and perspectives and therefore was well-aligned with the objectives of this study.^24,25^ The University of Pittsburgh Institutional Review Board deemed this study exempt.

### Participants and Recruitment

Clinician eligibility included 1) experience caring for children of caregivers who use LOE; 2) inability to speak the same language as ≥50% of caregivers who use LOE seen in their practice (eg, bilingual Arabic-speaking clinician who serves mostly Spanish-speaking population or solely English-speaking clinician); and 3) physician, nurse practitioner, or physician assistant. Caregiver eligibility criteria included 1) age 18 or older; 2) uses interpretation/translation services for their child(ren)’s medical visits; and 3) has used pediatric health care services for a child age 17 years or younger in the past year.

We recruited clinicians through email, listservs, and word of mouth from multiple inpatient and outpatient settings in a large mid-Atlantic children’s hospital. We recruited caregivers through direct referrals and from a list of caregivers who use LOE and had indicated interest in research opportunities. We used purposive sampling to recruit clinicians with diverse clinical specialties and caregivers with diverse language identities. After every 5 interviews, we reviewed the clinical specialties and caregiver languages of current participants and tailored recruitment to ensure representation (eg, emailing a specific clinical specialty). We also used snowball sampling, recruiting caregivers via direct referrals from clinicians who participated in the study. All participants received $50.

### Interview Guides and Surveys

Two team members developed separate clinician and caregiver interview guides (Appendix 1) with input from the full authorship team. Interview questions were aligned with our research questions and a review of the literature. Questions focused on 1) current use of AI language technologies in health care; 2) ways emerging AI can be used to achieve language equity; 3) concerns or challenges around using AI for language services; and 4) implementation and validation of AI technologies. We conducted an initial set of 9 interviews, after which we presented findings to an existing Immigrant & Refugee Health Committee of community experts. One team member was responsible for updating the guides in an iterative fashion based on questions and suggestions from this committee and the interviewers’ experiences. For example, we revised the language about AI to make it easier to understand due to respondents’ limited familiarity with AI language technologies apart from Google Translate. Surveys were administered to participants to collect relevant demographic information (Appendix 2). While we recognize that race, ethnicity, and gender are social constructs, we collected participants’ self-reported race, ethnicity, and gender as part of our survey given the impact of racism and gender discrimination on health care interactions and outcomes.^26^

### Data Collection and Analysis

We conducted 45- to 60-minute semistructured virtual interviews (Zoom or telephone) from May to August 2024 after obtaining verbal consent and completing a demographic survey. The interview team consisted of 6 medical students and researchers, all of whom were trained in qualitative research methods and supervised by an established physician-researcher with extensive qualitative research experience. We conducted clinician interviews in English and transcribed audio recordings verbatim. Fluent bilingual team members conducted caregiver interviews in Spanish, which were transcribed and analyzed in Spanish. Key quotations were translated into English for publication by a professional translation company (United Language Group) and then reviewed by a native Spanish speaker. For interviews with caregivers who spoke a language other than English or Spanish, we used telephonic interpretation (CyraCom). Only the English portions of these interviews were transcribed and analyzed. Interviews were coded iteratively and occurred concurrently with interviewing, allowing us to continue completing interviews until thematic saturation was reached and no new themes were heard.^25^

Data were coded and analyzed using thematic analysis.^25^ We uploaded deidentified interviews to Dedoose qualitative software and developed a codebook focused on clinician interviews with a list of a priori codes. Two study team members independently coded the first 6 clinician transcripts, adding new codes in an iterative fashion.^25^ Coders met weekly to identify emerging themes, resolve discrepancies, and add inductive codes. After achieving consistent coding, one of the team members coded the remaining interviews, with every third transcript co-coded. We discussed any discrepancies for co-coded transcripts and a third team member adjudicated if necessary. We coded caregiver interviews with the same approach, using the same codebook as similar codes emerged between caregivers and clinicians. Once all transcripts were coded, the research team met to finalize themes.

## Results

Twenty caregivers and 22 clinicians participated in this study. Clinicians were 86% female-identifying and 73% non-Hispanic White (Table 1). Exactly 70% of caregivers identified as female and 11 different languages were represented (Table 2). Five key themes with illustrative quotations are described below and in Table 3. While standard convention defines interpretation as spoken or signed communication and translation as written communication, participants sometimes used the terms interchangeably. We have preserved participants’ original word choices in all quotations.

### Theme #1: AI Language Technology is Used Primarily to Fill Professional Language Service Access Gaps

Participants reported that AI technology, specifically Google Translate, was used for written communication, verbal communication (interpretation) in emergencies when interpreters were not immediately available, or when there were issues with interpretation quality (eg, via telephone or video services).

Respondents described using Google Translate for written communication: “If the Epic reference is available, I will definitely use that… [if not] I’m using Google Translate and hoping that I have the ability for my interpreter to vouch [for] what I’m printing.”—Clinician#04 (Complex Care Pediatrician). Caregivers relied on Google Translate for postvisit instructions, preappointment forms, and patient portal messages: “…I look for the translator on Google and I take a photo of [the medicine instructions] and there it tells me how I have to give it to [my daughter], how many times, twice a day.”—Caregiver#08 (Spanish-speaking). “At the front desk, sometimes they give you some form to fill out… when there are things I don’t understand, that’s where I use Google Translation.”—Caregiver#16 (Kinyarwanda-speaking).

Caregivers and clinicians generally used telephonic interpretation for spoken interactions in health care, but reported using Google Translate for casual interactions or sometimes in emergencies: “My daughter had severe pain in her teeth, and I had to take her to the doctor. Because we couldn’t get an interpreter, I was putting everything in Google Translate… That was our only option.”—Caregiver#06 (Pashto-speaking).

In some instances, families and clinicians made a shared decision to switch to Google Translate when telephonic interpretation services were perceived to be inaccurate or connection was poor. A clinician shared a time when the family asked to turn off the interpreter service: “The interpreters, either we couldn’t hear them very well or they weren’t interpreting correctly, as in there was lots of repeated questions, lots of the answers didn’t make sense. I several times switched over to Google Translate…”—Clinician#16 (Intensive Care Unit Pediatrician).

### Theme #2: Caregivers and Clinicians Had Concerns About Using AI for Language Access in Health Care

Despite using Google Translate, caregivers and clinicians had concerns about using it or other AI platforms in health care settings. Subthemes included concerns about accuracy, privacy, and loss of human services. Accuracy was a major concern: “[If] I’m using Google Translate … I have to double-check it, triple-check it.”—Caregiver#17 (Uzbek-speaking). “The biggest [concern] is accuracy and trustworthiness of the translation…”—Clinician#05 (Pediatric Endocrinologist).

Respondents were also concerned about patient privacy, including information sharing, accessing AI on personal devices, and maintaining discretion in sensitive encounters: “…it needs to be encrypted. … how are we reaching out to virtual information, and is that transmission protected? … We really got to think about how are we making that translation through the air safe.”—Clinician#10 (Adolescent Medicine Nurse Practitioner).

Caregivers and clinicians worried that the availability of AI would lead to loss of human services: “Now that we are sort of shifting to use AI, I have a general concern that [interpreter services] may reduce their service or reduce the number of interpreters.”—Clinician#03 (Adolescent Medicine Nurse Practitioner). A caregiver worried about the capabilities of AI compared to humans: “…maybe the language service technology…wouldn’t render a word or interpretation for a word the same way a human interpreter would, or accurately, as a human interpreter might. Maybe they’re just not as competent…in interpreting my language as much as a human interpreter.”—Caregiver#03 (Arabic-speaking). Participants also emphasized empathy and body language as critical parts of clinician-family interaction that could be lost with AI: “Maybe for human is better…AI is cold…but human is warm.”—Caregiver#01 (Mandarin-speaking). “There’s body language…I think the interpreter can probably get a better sense of what the person’s actually trying to say and the tone…the meaning comes across more clearly when it’s in person.”—Clinician#05 (Pediatric Endocrinologist).

### Theme #3 AI Has Potential to Improve Real-Time Translation and Interpretation

Caregivers and clinicians were open to using AI for translation and interpretation in the future, despite their misgivings. Subthemes included using AI for written translation, making verbal communication more convenient, and in select cases using AI for sensitive topics. Participants wanted to use AI for automatic, real-time translation of written documents: “… if you can scan a document…and it can just reprint it in their language…consents, things like that, or discharge instructions or medications…if it was very quick and able to do that…that would be amazing.”—Clinician#09 (Same-Day Surgery Physician Assistant). Another clinician shared: “I do think Google Translate or something like that could be an option as far as written material…the after-visit summary or when we’re sending out [patient portal] message…”—Clinician#14 (Community Pediatric Nurse Practitioner).

Caregivers imagined using AI for verbal communication with staff during hours when live, video, and telephonic interpreters were perceived as less available (eg, evenings, weekends) or for quick communication (eg, simple medical questions or nonmedical communication) where getting an interpreter was thought to be cumbersome: “[I would use AI] When I need to communicate with the nurses.”—Caregiver#15 (Russian-speaking). In addition to more efficient interpretation, participants hoped AI could better accommodate languages and dialects: “People speaking languages that are less commonly spoken than Spanish or…specific regional dialects…there may only be one interpreter who the family feels comfortable that they’re actually translating or interpreting correctly. And that’s something that is always on my mind when there’s a switch [in interpreter] midway through because of a technical issue.”—Clinician#07 (Pediatric Hospitalist).

Surprisingly, a few participants imagined AI could make discussing sensitive topics easier. One caregiver shared: “I think sometimes people maybe is comfortable with no person there…maybe AI is better. If AI is good enough for translation, I think people maybe feel more comfortable…”—Caregiver#01 (Mandarin-speaking). However, participants hoped AI could still respond to body language cues and facial expressions: “[Ideally AI would be] translating word for word…able to pick up on minute things like body language and inflection of your voice and speed of your speech.”—Clinician#11 (Palliative Care Physician Assistant).

### Theme #4 Ethical AI Use Requires Equitable Development and Access for Both Patients and Clinicians

Participants emphasized the importance of equity and accessibility in AI development. Caregivers stressed the importance of accessible AI technology that could overcome literacy barriers: “[some] don’t know how to read [or] write in our language, so they don’t know how to use Google Translate. If they could speak… and then it turns it into English, or when it’s in English, it turns into Pashto, that would make it easier.”—Caregiver#05 (Pashto-speaking).

Participants wanted AI to be easy to use regardless of experience with technology, particularly as most caregivers were not familiar with AI beyond Google Translate: “I am not computer savvy…I am able to use a basic application like…to log into Zoom…But nothing more than that.”—Caregiver#02 (Dari-speaking).

Clinicians expressed concerns about bias in AI training data, which could compromise equity and cultural sensitivity: “As we’ve seen that it’s learning on what’s already available, and that is racist, misogynist. There’s lots of stuff out there that isn’t great…And you can see that when you put in a case, it is biased…I worry about that a lot.”—Clinician#16 (Intensive Care Unit Pediatrician).

When considering ease of access during clinical visits, clinicians recommended building AI to be easily accessible on their phone or the electronic medical record: “If it were built into Epic and you could use it without having to call a third party, so you open a video visit and there is AI interpretation already there.”—Clinician#06 (Community Pediatrician). Another clinician shared: “It would be something like you could have on your phone…really accessible.”—Clinician#13 (Neuro-Oncology Physician Assistant).

### Theme #5: Additional Validation and Input From Community Members, Clinicians, and Leaders Are Needed to Feel Comfortable Using AI

Participants asked for reliability studies to ensure AI was accurate before use: “I would first want to make sure it would be validated…Probably standardized patient encounters to make sure that the efficiency and the precision of the AI, it would match a medically trained in-person interpreter.”—Clinician#19 (Pediatric Orthopedic Surgeon). A caregiver shared: “We might need to do comparison, so to check whether the Google Translate is accurate or not… I cannot tell whether it’s accurate or not.”—Caregiver#01 (Mandarin-speaking).

Clinicians wanted to know the technology was endorsed by hospital leadership, other clinicians, language experts, and families: “…would have to come from both healthcare system and hospital leadership…it would be valuable to get buy-in from multidisciplinary sources, like folks within our language services communities, international communities.”—Clinician#07 (Pediatric Hospitalist). Another clinician shared: “What would really probably get me the most excited is…if I heard it from families…that it changed their stress while in the hospital…that would really get me to be on board.”—Clinician#22 (Pediatric Nephrologist).

### Caregiver Versus Clinician Views

Caregivers and clinicians had similar opinions on the use of AI, though clinicians were more aware of emerging technologies in general (eg, ChatGPT). Both groups were most familiar with Google Translate. There was similar emphasis on the need to maintain accuracy, privacy, empathy, and human translation and interpretation services if AI is used. Caregivers were more likely to report using Google Translate for practical, immediate needs and day-to-day use such as filling out forms, understanding instructions, using the patient portal, and sometimes in emergencies. Clinicians focused more on logistics of use (eg, integration into the medical record vs an app for download on a personal device), requirements for formal validation studies, and equitable creation and access to the technology.

## Discussion

In this qualitative study examining perspectives of caregivers and clinicians about the use of AI to provide language services in health care, respondents were open to using AI to fill gaps in translation and interpretation. However, they preferred human services and wanted to see validation and significant buy-in from patients, hospital leadership, and language experts before use. Responsible use of AI language technology offers an opportunity to foster trust between caregivers who use LOE and clinicians by expanding equitable communication. However, using AI before it is ready may introduce potential for a higher rate of medical errors and exacerbate existing health care inequities for people who use LOE.^27,28^ Caregivers and clinicians strongly emphasized the value of human services—either in person or via video or telephone—to provide context, clarification, support, and a “human touch.” Prior studies have highlighted the importance of meaning conveyed through body language and facial expressions, further emphasizing the importance of in-person human interpretation.^29,30^ Results from our study suggest that hospital systems must invest in comprehensive language services, including validation of AI technology and recruitment, retention, and thriving of human interpreters and translators.

Using AI for real-time translation of documents was a potential use that emerged, consistent with studies highlighting limited access to translation services (eg, discharge instructions).^31,32^ Patients often use unvalidated and error-prone methods to translate documents at home, such as Google Translate or relying on friends or family.^33^ Health care systems have increasingly included more written communication, especially since the shift to virtual care in the wake of the COVID-19 pandemic and the 21st Century Cures Act, which prioritizes patient access to their medical documents.^34^ Patients who use LOE are less able to access telemedicine services^35^ and less likely to access their medical records or the patient portal.^36^ A recent study evaluating the frequency of patient portal translation into LOE found that of the 448 portals reviewed, 66% were available in a LOE, though only 23% were available in a language other than English or Spanish. Even for portals available in LOE, all portal components were not translated consistently.^37^ Lack of real-time translation of patient messages effectively excludes individuals who use LOE from a convenient, confidential method of accessing care. Using validated AI technologies for rapid, real-time translation in conjunction with certified translators could help address this disparity.

Lack of interpretation services in emergency situations was brought up by participants as an ongoing inequity in health care. Studies have shown that interpreter services are underused in emergency or high-acuity settings,^38^ which may lead to higher revisit and medical error rates.^39^ Caregivers reported relying on Google Translate in the emergency room, and clinicians highlighted the importance of real-time language services given the need for accurate, expeditious, and sensitive communication around health care decisions and outcomes. While AI is a tempting “quick fix,” efforts should instead focus on strengthening on-demand human interpretation and translation services for these high-acuity situations. Use of language justice approaches, whereby language services are seamlessly embedded in clinical workflows and which include comprehensive training for all staff, may be a helpful approach to address inequities in emergency settings.^11^

Appropriate use of AI also requires that caregivers and clinicians are knowledgeable about evolving technology. It was notable that despite rapid movement of AI toward large language models such as ChatGPT, most caregivers and clinicians had limited familiarity with AI that could be used for translation and interpretation other than Google Translate. While we attempted to ask about AI for translation or interpretation more generally, we often needed to narrow our discussion to Google Translate as a familiar example. This discrepancy between the rapid development of AI and clinician and caregiver understanding highlights the importance of accessibility and developing engaging ways for clinicians and families to learn how to best use AI. Clinicians were more familiar with the concept of AI than most caregivers, underscoring the need to address linguistic and socioeconomic barriers to accessing new technology, including digital literacy, access to devices, and multilingual user interfaces. Future studies should explore the use of AI for translation and interpretation (eg, having participants use different AI language technologies in real time).

The study was limited to 1 institution that is part of a large health care system. Findings may not be applicable to other health systems with different access to translation and interpretation resources, although the goal of qualitative research is not to generalize but to capture participants’ stories and perspectives. A key strength of this study was that we included caregivers using a wide variety of languages, important as most research in LOE includes only Spanish.^40^ However, to do so, we used telephonic interpreters and included in the analysis only the interpreted, English-language parts of the transcripts. Our interviews also did not include perspectives of language service providers or technology specialists, whose future input on what is possible and how to achieve it will be critical.

Responsible use of AI language technologies requires careful, multilevel efforts to ensure technologies are trustworthy, strengthen patient-provider relationships, and improve patient outcomes.^27^ Future research should focus on the validation of evolving technologies for all languages. From a clinical perspective, patients and caregivers should be involved in decisions about the types of language services used,^11^ and clinicians should continue to learn and be open to new technologies if there is potential for improved patient care. Hospitals and health care systems must establish evidence-based guidelines for ethical, practical use of AI and undertake systems-level change to ensure accurate, equitable language services at all encounters regardless of urgency or acuity. Initiatives such as the Stakeholders Advocating for Fair and Ethical AI in Interpreting Task Force, Collaborative Assessment for Responsible and Ethical AI Implementation model, and the development of an ethics checklist for using AI in health care aim to establish guidance for responsible use.^41^ Nonetheless, Section 1557 of the Affordable Care Act requires that translation be provided by a qualified translator, thus automated translation alone is not currently sufficient to meet legal standards.^42^ Policies should focus on elevating the role of human interpreters and translators in medical care and investing in systems-level solutions to seamlessly integrate language services into all aspects of pediatric health care.^11^

## Conclusion

Caregivers and pediatric clinicians were open to using AI to improve communication, but had concerns about accuracy, privacy, loss of human services, empathy, and equity. As AI technologies evolve, we must find a delicate balance between technological advancement and patient-centered care. It is critical to expand and allocate resources toward human translation and interpretation, working toward a future in which AI is a tool to enhance equitable translation and interpretation alongside human services.

## Supplementary Material

1

2

Supplementary data associated with this article can be found in the online version at doi:10.1016/j.acap.2025.102887.

## Figures and Tables

**Table 1.** Clinician Demographics (n = 22)

Demographic	# Clinician Responses (% of Total Clinicians)*	

Clinician type	Physician	12 (55%)
	Nurse practitioner	6 (27%)
	Physician assistant	4 (18%)
Specialty	General outpatient	7 (32%)
	Emergency	5 (23%)
	Medical subspecialist	5 (23%)
	Surgery	3 (14%)
	General inpatient	2 (9%)
Racial and ethnic background	Asian	1 (5%)
	Black, African, or African American	1 (5%)
	Hispanic, Latino, Latina, Latine, or Latinx	1 (5%)
	Middle Eastern or North African	2 (9%)
	White or Caucasian	16 (73%)
	Other^†^	1 (5%)
Gender identity	Cisgender female/woman	19 (86%)
	Cisgender male/man	3 (14%)
Percent of time in clinical practice (%)	76–100	12 (55%)
	51–75	6 (27%)
	26–50	2 (9%)
	25	2 (9%)
Length of time in practice (y)	1–5	4 (18%)
	6–10	10 (46%)
	11–15	3 (14%)
	16–20	2 (9%)
	21–25	2 (9%)
	25–30	1 (5%)
Average percent of patients who primarily speak LOE^‡^ (%)	1–5	10 (46%)
	6–10	5 (23%)
	11–15	3 (14%)
	16–20	3 (14%)
	21–25	0 (0%)
	26–30	1 (5%)

LOE indicates languages other than English.

* Percentages may not add to 100% due to rounding.

† Other included 1 individual who self-described their racial and/or ethnic background as “Jewish.”

‡ Clinicians self-reported percent of patients who primarily speak LOE and thus data may be subject to recall bias.

**Table 2.** Caregiver Demographics (n = 20)

Demographics	# Caregiver Responses (% of Total Caregivers)*	

Language	Arabic	1 (5%)
	Dari	2 (10%)
	Kinyarwanda	1 (5%)
	Mandarin	1 (5%)
	Nepali	1 (5%)
	Pashto	3 (15%)
	Portuguese	1 (5%)
	Russian	2 (10%)
	Spanish	5 (25%)
	Swahili	1 (5%)
	Uzbek	2 (10%)
Age (y)	21–30	5 (25%)
	31–40	9 (45%)
	41–50	5 (25%)
	51–60	0 (0%)
	61–63	1 (5%)
Racial and ethnic background	Asian	9 (45%)
	Black, African, or African American	1 (5%)
	Hispanic, Latino, Latina, Latine, or Latinx	3 (15%)
	Middle Eastern or North African	1 (5%)
	White or Caucasian	1 (5%)
	Other^†^	2 (10%)
	No response	3 (15%)
Gender^‡^	Female	14 (70%)
	Male	6 (30%)

* Percentages may not add to 100% due to rounding.

† Caregivers were asked to self-describe their racial and ethnic background. Most caregivers identified within our predefined groups, but 1 participant self-described specifically as “Nubian” and another as “Uzbek.”

‡ Caregivers were asked to self-describe their gender.

**Table 3.** Additional Representative Quotations

Key Finding		Quotation

Theme #1: AI language technology is used primarily to fill professional language service access gaps	Subtheme #1A: Written communication	“I have to go into Google, put the translator there there and it is like taking a picture... and from there it translates and with that I understand it.”—Caregiver#09 (Spanish-speaking) “In case something [I don’t] understand, then [I use] Google Translate.”—Caregiver#11 (Swahili-speaking) “When I have to sign consent, I use Google Translate, and [it] is enough for me.”—Caregiver#15 (Russian-speaking)
	Subtheme #1B: Verbal communication	“Yes, it’s very useful. In fact, I used [Google Translate] quite a bit with the nurses. It was an easy tool to access because I had it in my pocket... it is not perfect, but it works.”—Caregiver#14 (Spanish-speaking) “Sometimes I have to record on Google Translate before making the phone call, the information that I want to render to them. And then once they respond, I just play that recording. It’s not easy.”—Caregiver#17 (Uzbek-speaking) “...but let’s say the patient is on the floor. There’s no interpreter 24/7... for more critical conversations, we always try to have the in-person interpreter. But for things like, ‘How do you feel?’ like, ‘Are you feeling okay today?’ or you’re just having a quick check-in, you could do like we’ve been doing on our phones, like some Google Translate.”—Clinician#17 (Pediatric Cardiologist)
	Subtheme #1C: Issues with interpreter	“They always try to provide us with an interpreter whenever we go to the doctor’s appointment, it’s that sometimes the connection problems...sometimes the interpreter gets disconnected, and then there is an issue reconnecting. In those cases, we’ve used Google Translate.”—Caregiver#17 (Uzbek-speaking) “The only thing that I would like is that during nighttime, during non-waking hours, or during the middle of the night, so that it could get in touch with the interpreter faster.”—Caregiver#18 (Russian-speaking) “...sometimes there are a few of them... that do not relay information fully. And when I try to explain something in detail about my child’s condition to the doctor, the interpreter asks me to speak in shorter sentences, but I want to give those details to the doctor. So I don’t feel okay to shorten those sentences.”—Caregiver#20 (Uzbek-speaking)
Theme #2: Caregivers and clinicians had concerns about using AI for language access in health care	Subtheme #2A: Accuracy	“If there is a potential for that kind of [accuracy] error, then that doesn’t actually help us... that makes it harder for us... our hope and our effort would be to make such a machine or such technology that would have zero errors so that it would help human beings rather than make things further complicated for them.”—Caregiver#06 (Pashto-speaking) “[It is important] that the translation is accurate ... and that there’s not errors...I think there definitely could be a role for it. I think it’d just have to be well-tested, well-vetted.”—Clinician#12 (Rehabilitation Nurse Practitioner) “I think accuracy is always the thing with the AI. Right now, what you can see with ChatGPT, the accuracy seems pretty good. And same with the Google Translate. But I think that will be my number one concern.”—Clinician#17 (Pediatric Cardiologist) “Accuracy [is a concern]. A lot in language can be lost in lack of tone or understanding of subtlety in accent and language that I think translation loses. Cultural implications of using certain terminology can be lost and lead to miscommunication.”—Clinician#20 (Cardiovascular Intensive Care Unit Pediatrician)
	Subtheme #2B: Privacy	“So I usually don’t share a lot of things to internet. But I know that, for example, if providers have my social security number or my other information is protected... But other applications ... I usually do not use or share [on] my phone.”—Caregiver#04 (Pashto-speaking) “I think for sensitive cases, I think for sexual assault or potentially anything that might involve ... non-accidental traumas. Those are ones that I probably would have some hesitation ... There’s some part of you that’s always hesitant to record people.”—Clinician#15 (Emergency Medicine Physician Assistant) “These corporations that are working on [AI] are clearly interested in more and more information. So how that information is used in later applications is always a concern.”—Clinician#20 (Cardiovascular Intensive Care Unit Pediatrician)
	Subtheme #2C: Loss of human services	“I think the best thing, especially for my daughter, who’s nonverbal, is to be able to see who she’s interacting with... if they could ask her, ’Where is it hurting?’ and she could point it out ... And not just hearing, in a setting where she could see the provider and the provider could see her, I think that would be very helpful.”—Caregiver#06 (Pashto-speaking) “I hope it continues... that translators continue to exist in hospitals.... It’s a great tool.”—Caregiver#07 (Spanish-speaking) “The reason why I sometimes feel that maybe in-person interpreter would be better is because I remember the situation where it’s so much easier for me to have a Nepali doctor and to communicate straight where you can see face-to-face. That’s the only time where I feel like it would be better if I have a Nepali in-person interpreter.”—Caregiver#10 (Nepali-speaking) “I am afraid of [AI] changing what I want to say. And if there is an interpreter, it’s easier for me to explain.”—Caregiver#13 (Portuguese-speaking) “...having a human who can kind of go back and forth with that person ... I think that would be hard with AI... that human connection with someone who understands your language, it just is hard to substitute that... the type of situations where you’re talking about we do in the ICU that are so intense that families remember forever. Just having a human say those things, I think, is so much more important.”—Clinician#22 (Pediatric Nephrologist)
Theme #3 AI has potential to improve real-time translation and interpretation	Subtheme #3A: Written document translation	“It would be nice to message back and forth...Because *via* messages, it’s easier to understand what they say, what they’re talking about, and it’s also easier ... and faster to use [an automated] translator.”—Caregiver#18 (Russian-speaking) “The most common diagnoses are the only [resources] available. And then the less common languages, even the most common things aren’t available in those languages, so it’s hard to give the typical instructions... we don’t really have that large of a Spanish-speaking population. We just have this smattering of other languages, so it’s really hard to know what to do.”—Clinician#06 (Community Pediatrician) “I think it would be more accurate [than Google Translate]. At least I would hope it would be more accurate information. I think it would be... I think it would be readily available, easily available. Accurate information given to the patient. I think it would be convenient.”—Clinician#08 (Inpatient Nurse Practitioner) “Consents really should be translated... I would need them to be quick... this can’t be something that you request the [translation] and you get an hour later. It has to be instant for our pace of work in the ER.”—Clinician#18 (Emergency Medicine Pediatrician)
	Subtheme #3B: Verbal communication	“I hope that artificial intelligence improves... you can take very, very good advantage of it, because it has many advantages, such as privacy, which is very true, and availability... because when you want an interpreter in person, you have to schedule it, it’s not so immediate ... with enough time in advance... if I have some kind of emergency, if I want to go today...The immediacy of being able to access that service... it is also an advantage of using this artificial intelligence equipment.”—Caregiver#14 (Spanish-speaking) “Especially on weekends, either Saturday or Sunday. At the ER... it’s very difficult for them to find interpreters.”—Caregiver#19 (Dari-speaking) “Not just less common languages, but in general, there’s always an issue in either the parent having a hard time or us having a hard time, interpreter having a hard time. We are always frustrated [with the audio quality of current phone and video interpretation].”—Clinician#03 (Adolescent Medicine Nurse Practitioner) “It would probably be helpful to have an animated face to go along with it that might be able to demonstrate ... culturally appropriate facial expressions ... In some cultures, the shake of the head means something different.”—Clinician#19 (Pediatric Orthopedic Surgeon)
	Subtheme #3C: Sensitive topics	“... a situation of private things ... when they put the translator with video call, what I do is that I turn it to the other side, I don’t talk directly to the person behind you because I kind of find that... saying things like that, kind of private... sometimes I have been reluctant to say it and that is why it is better when I go to my doctor, because I tell him things there because it is directly to him.”—Caregiver#12 (Spanish-speaking) “...there are things that culturally you are predisposed to possibly be embarrassed about, I don’t know, to talk about things that are very intimate and if there is an interpreter present or well, it can be uncomfortable...say you are in a consultation and you have to undress or you have to show something, then you can feel uncomfortable that the interpreter turns around ... on the other hand with an artificial device, with someone who is not human, well, it would be better.”—Caregiver#14 (Spanish-speaking) “Those may be times where it would be nice to be able to speak into a box...there’s not somebody else on the other end.”—Clinician#02 (Community Pediatrician)
Theme #4 Ethical AI use requires equitable development and access for both patients and clinicians		“Accessibility, because to use AI, you are assuming that the person has access to some type of technology... I would want to make sure that there is fair access to all of it.”—Clinician#11 (Palliative Care Physician Assistant) “Ease of access such that every team member would not find it to be burdensome to use.”—Clinician#18 (Emergency Medicine Pediatrician) “[It would be easy] If there could be a way that you can input sort of similar to the way Google Translate currently has, where you can use the lens in your phone... where you can scan documents and have them translated ... into multiple languages and then have it be available. And you could just click a button and say, ’Print in X [language],’ as opposed to translating it each time separately.”—Clinician#20 (Cardiovascular Intensive Care Unit Pediatrician) “... something that we could click on ... that popped up and we could exchange information real time right there.”—Clinician#21 (Heart Failure and Transplant Nurse Practitioner)
Theme #5: Additional validation and input from community members, clinicians, and leaders are needed to feel comfortable using AI		“...making sure they have a validation for the service...the service needs to have spot checks ... and also some ability to have a fallback plan if it doesn’t seem to be working.”—Clinician#01 (Pediatric Endocrinologist) “It would be helpful to see feedback from the families to hear them say how well they thought that the devices worked and maybe even like a third-party validation... a Nepalese interpreter that could say, “Yes, this AI translated the information correctly.”—Clinician#11 (Palliative Care Physician Assistant) “I would need some type of tool, study, randomized controlled trial... that was able to validate the conversations that were being had so that I would know that they were being accurately translated.”—Clinician#11 (Palliative Care Physician Assistant) “...we’ll have to, as a community, come up with a process to validate all these new tools. So that process could be that five native-speaking physicians or people who speak both languages have looked into the translations and felt it’s accurate.”—Clinician#17 (Pediatric Cardiologist) “... if we’re using the voice recording systems, it has to be validated for not just men, but also women’s voices and also young and old people’s voices. Because some concern, at least, about these dictation systems in the past is that a lot of it was validated with men’s voices, not female voices, so they don’t work very well for women.”—Clinician#17 (Pediatric Cardiologist) “...with all of these emerging technologies, regulation on data use and involvement of patient family advocacy groups ... would be great to maintain responsibility.”—Clinician#20 (Cardiovascular Intensive Care Unit Pediatrician) “...any attending physician that’s providing day-to-day, inpatient or outpatient patient care should have a voice... there’s an army of advanced practice providers, nurse practitioners, physician assistants that we’re either standing with that attending physician, providing the care, or frankly, provide a lot of care independently too... But you know you shouldn’t and can’t exclude the bedside nurse or respiratory therapist.”—Clinician#21 (Heart Failure and Transplant Nurse Practitioner)

## What’s New

Caregivers and clinicians viewed artificial intelligence as promising to fill gaps in health care translation and interpretation, but were concerned about accuracy, privacy, loss of human services, and equity. Further research on responsible practice and policy is needed.

## References

[1] Civil Rights Division | Executive Order 13166 Limited English Proficiency Resource Document: Tips and Tools from the Field. Available at: https://www.justice.gov/crt/fcs/EO13166_tips. Accessed December 19, 2024.

[2] DeCamp LR, Choi H, Davis MM. Medical home disparities for Latino children by parental language of interview. J Health Care Poor Underserved. 2011;22:1151–1166. 10.1353/hpu.2011.0113 22080700 PMC4506841

[3] Flores G, Tomany-Korman SC. The language spoken at home and disparities in medical and dental health, access to care, and use of services in US children. Pediatrics. 2008;121:e1703–e1714. 10.1542/peds.2007-2906 18519474

[4] Batalova J, Zong J. Language diversity and English proficiency in the United States. Migration Information Source. November 11, 2016. Available at: https://www.migrationpolicy.org/article/language-diversity-and-english-proficiency-united-states. Accessed December 19, 2024.

[5] Woods T, Hanson D. Over half of children of immigrants are bilingual. Urban Wire. Urban Institute. October 18, 2016. Available at: https://www.urban.org/urban-wire/over-half-children-immigrants-are-bilingual. Accessed December 19, 2024.

[6] Yeboah D, McDaniel C, Lion KC. Language matters: why we should reconsider the term “limited English proficiency”. Hosp Pediatr. 2023;13:e11–e13. 10.1542/hpeds.2022-007014 36464981

[7] Quan K, Lynch J. The High Costs of Language Barriers in Medical Malpractice. California: National Health Law Program; 2010.

[8] Khan A, Yin HS, Brach C, Association between parent comfort with English and adverse events among hospitalized children. JAMA Pediatr. 2020;174:e203215. 10.1001/jamapediatrics.2020.3215 33074313 PMC7573792

[9] Yin HS, Mendelsohn AL, Wolf MS, Parents’ medication administration errors: role of dosing instruments and health literacy. Arch Pediatr Adolesc Med. 2010;164:181–186. 10.1001/archpediatrics.2009.269 20124148

[10] Karliner LS, Pérez-Stable EJ, Gregorich SE. Convenient access to professional interpreters in the hospital decreases readmission rates and estimated hospital expenditures for patients with limited English proficiency. Med Care. 2017;55:199–206. 10.1097/MLR.0000000000000643 27579909 PMC5309198

[11] Ragavan MI, Méndez DD, Caballero TM. Promoting language justice for children with medical complexity and their families: an urgent call to action. Hosp Pediatr. 2024;14:e358–e361. 10.1542/hpeds.2024-007792 39069818 PMC11287058

[12] Rainer MF Re: Language Access Provisions of the Final Rule Implementing Section 1557 of the Affordable Care Act. U.S. Department of Health and Human Services, Office for Civil Rights; 2024.

[13] Alvarado-Little W, Flores G. Language matters: language inclusivity in pediatric health care. Acad Pediatr. 2024;24(7S):S217–S219. 10.1016/j.acap.2023.11.022 39428159

[14] Noll R, Frischen LS, Boeker M, Machine translation of standardised medical terminology using natural language processing: a scoping review. N Biotechnol. 2023;77:120–129. 10.1016/j.nbt.2023.08.004 37652265

[15] Khoong EC, Steinbrook E, Brown C, Assessing the use of Google Translate for Spanish and Chinese translations of emergency department discharge instructions. JAMA Intern Med. 2019;179:580–582. 10.1001/jamainternmed.2018.7653 30801626 PMC6450297

[16] Thirunavukarasu AJ, Ting DSJ, Elangovan K, Large language models in medicine. Nat Med. 2023;29:1930–1940. 10.1038/s41591-023-02448-8 37460753

[17] Nasution AH, Onan A. ChatGPT label: comparing the quality of human-generated and LLM-generated annotations in low-resource language NLP tasks. IEEE Access. 2024;12:71876–71900. 10.1109/ACCESS.2024.3402809

[18] Gimeno A, Krause K, D’Souza S, Completeness and readability of GPT-4-generated multilingual discharge instructions in the pediatric emergency department. JAMIA Open. 2024;7:ooae050. 10.1093/jamiaopen/ooae050 38957592 PMC11216721

[19] Zhang M A study on the translation quality of ChatGPT. IJECMR. 2024;5:153–163. 10.38007/IJECMR.2024.050121

[20] Siu SC. ChatGPT and GPT-4 for professional translators: exploring the potential of large language models in translation. SSRN J. 2023:1–36. 10.2139/ssrn.4448091

[21] Panayiotou A, Hwang K, Williams S, The perceptions of translation apps for everyday health care in healthcare workers and older people: a multi-method study. J Clin Nurs. 2020;29:3516–3526. 10.1111/jocn.15390 32558965

[22] Scott IA, Carter SM, Coiera E. Exploring stakeholder attitudes towards AI in clinical practice. BMJ Health Care Inform. 2021;28:e100450. 10.1136/bmjhci-2021-100450 PMC8663096 34887331

[23] Meskó B The impact of multimodal large language models on health care’s future. J Med Internet Res. 2023;25:e52865. 10.2196/52865 37917126 PMC10654899

[24] Kahlke RM. Generic qualitative approaches: pitfalls and benefits of methodological mixology. Int J Qual Methods. 2014;13:37–52. 10.1177/160940691401300119

[25] Patton MQ. Qualitative Research & Evaluation Methods: Integrating Theory and Practice. Google Books. Available at: https://tinyurl.com/bdf5efhv. Accessed August 7, 2024.

[26] Heidari S, Babor TF, De Castro P, Sex and gender equity in research: rationale for the SAGER guidelines and recommended use. Res Integr Peer Rev. 2016;1:2. 10.1186/s41073-016-0007-6 29451543 PMC5793986

[27] Lion KC, Lin Y-H, Kim T. Artificial intelligence for language translation: the equity is in the details. JAMA. 2024;332:1427–1428. 10.1001/jama.2024.15296 39264601

[28] Brewster RCL, Gonzalez P, Khazanchi R, Performance of ChatGPT and Google Translate for pediatric discharge instruction translation. Pediatrics. 2024;154:e2023065573. 10.1542/peds.2023-065573 38860299

[29] Gerwing J, Li S. Body-oriented gestures as a practitioner’s window into interpreted communication. Soc Sci Med. 2019;233:171–180. 10.1016/j.socscimed.2019.05.040 31203145

[30] Maletsky KD, Worsley D, Tran Lopez K, Communication experiences of caregivers using a language other than English on inpatient services. Hosp Pediatr. 2023;13:471–479. 10.1542/hpeds.2022-007011 37125497

[31] Davis SH, Rosenberg J, Nguyen J, Translating discharge instructions for limited English-proficient families: strategies and barriers. Hosp Pediatr. 2019;9:779–787. 10.1542/hpeds.2019-0055 31562199 PMC6876295

[32] Isbey S, Badolato G, Kline J. Pediatric emergency department discharge instructions for Spanish-speaking families: are we getting it right? Pediatr Emerg Care. 2022;38:e867–e870. 10.1097/PEC.0000000000002470 34140448

[33] Corona R, Stevens LF, Halfond RW, A qualitative analysis of what Latino parents and adolescents think and feel about language brokering. J Child Fam Stud. 2012;21:788–798. 10.1007/s10826-011-9536-2

[34] Individuals’ access and use of patient portals and smartphone health apps. HealthIT.gov; 2022. Available at: https://www.healthit.gov/data/data-briefs/individuals-access-and-use-patient-portals-and-smartphone-health-apps-2022. Accessed January 6, 2025.

[35] Chang E, Davis TL, Berkman ND. Differences in telemedicine, emergency department, and hospital utilization among nonelderly adults with limited English proficiency Post-COVID-19 pandemic: a cross-sectional analysis. J Gen Intern Med. 2023;38:3490–3498. 10.1007/s11606-023-08353-7 37592119 PMC10713935

[36] El-Toukhy S, Méndez A, Collins S, Barriers to patient portal access and use: evidence from the health information national trends survey. J Am Board Fam Med. 2020;33:953–968. 10.3122/jabfm.2020.06.190402 33219074 PMC7849369

[37] Prada-Rey N, Samal L, Rodriguez JA. Language availability of patient portals at academic medical centers in the United States. J Gen Intern Med. 2025;40:1477–1479. 10.1007/s11606-024-09180-0 39500842 PMC12045902

[38] Lion KC, Gritton J, Scannell J, Patterns and predictors of professional interpreter use in the pediatric emergency department. Pediatrics. 2021;147:e20193312. 10.1542/peds.2019-3312 33468598 PMC7906072

[39] Portillo EN, Stack AM, Monuteaux MC, Association of limited English proficiency and increased pediatric emergency department revisits. Acad Emerg Med. 2021;28:1001–1011. 10.1111/acem.14359 34431157

[40] Chen A, Demaestri S, Schweiberger K, Inclusion of non-English-speaking participants in pediatric health research: a review. JAMA Pediatr. 2023;177:81–88. 10.1001/jamapediatrics.2022.3828 36315130 PMC10854994

[41] Interpreting SAFE AI Task Force. Guidance on AI and Interpreting Services. June 2024. Available at: https://safeaitf.org/guidance/#elementor-toc__heading-anchor-19. Accessed January 11, 2025.

[42] Federal Register, Volume 81 Issue 96 (Wednesday, May 18, 2016). Available at: https://www.govinfo.gov/content/pkg/FR-2016-05-18/html/2016-11458.htm. Accessed May 19, 2025.

