# Supplementary material for: Caregiver and Pediatric Clinician Perspectives on Artificial Intelligence for Language Services
Source: Acad Pediatr. Author manuscript; Available in PMC 2026 Jul 7. (PMC13341081; doi:10.1016/j.acap.2025.102887)
Supplement: 1 [file NIHMS2170835-supplement-1.docx]

**Supplemental Appendix 1. Initial Version of Interview Guides**

**Clinician Interview Guide**

***Translation Current Practices***

*We would like to ask you some targeted questions about* ***translation*** *services (i.e., services used for* ***written*** *communication with patients in a language other than English). Examples include written medication or discharge instructions, consent forms, electronic health record / chart notes, patient portal messages, etc.*

1. Please tell me about how you communicate in written form with people who speak a language other than English. *Probe on the use of pre-translated documents in the EMR, in-person or virtual interpreters (iPad vs. Telephone), Google Translate, family members, office staff who speak the patient’s language, etc.*
   1. What kinds of challenges or frustrations do you and your patients face when communicating in written form? *Probe on how this may differ between more common vs. less common LOE in our area*. *Probe on real-time translation (e.g., discharge instructions) and information for patient reference at home (e.g., medication dosing)*

***Interpretation Current Practices***

*We would now like to ask you some more targeted questions about* ***interpretation*** *services (i.e., services used to* ***verbally*** *communicate with patients in a language other than English). Examples include a patient encounter in the hospital or office visit, telehealth visit, or phone call.*

1. Please tell me about how you communicate verbally with people who speak a language other than English. *Probe on the use of in-person or virtual interpreters (iPad vs. Telephone), Google Translate, family members, office staff who speak the patient’s language, etc.*
   1. What kinds of challenges or frustrations do you and your patients face when communicating verbally? *Probe on how this may differ between more common vs. less common LOE in our area*.

*Now, we will ask you some questions about artificial intelligence language technologies. Artificial intelligence is defined as computer systems that perform tasks that normally require human intelligence and can be used in a very wide range of settings. Note we are not necessarily promoting or discouraging use of these technologies, we are interested in learning from your perspectives.*

***Current Use of Language Technologies***

1. How, if at all, have you used these technologies in healthcare settings?
   1. What do you like about using it / what are its strengths?
   2. What are your concerns about it / barriers to using it?

***Opinion on Emerging AI Language Technologies (15 min)***

*Now, we are going to shift to more emerging technologies, i.e., technologies beyond Google Translate that may or may not be widely used or known about at this point.*

*NOTE FOR INTERVIEWER: For the following questions, Allow the interviewee to come up with ideas first without prompting with examples*. *If not mentioned or interviewee unsure, probe on their awareness of ChatGPT and its translation capabilities as an example. Remind the interviewee that we do not need to know if the technology is fully accurate or not we are mainly trying to generate ideas and gage awareness.*

1. What emerging AI technologies, if any, have you seen or heard of being used for medical document translation? For medical interpretation?
2. How would you imagine using new AI language technologies in your clinical practice? *Probe on utilization overseen by human interpreters/translators. Probe on supplementing human translation, real-time translation in a discharge setting, translation of documents for later reference, etc.*
   1. What possibilities are you excited about, if anything?
   2. What concerns do you have, if any? *Probe on how this could vary depending on clinical situation (e.g., medication instructions, surgery consent, delivering a diagnosis of a serious illness)*
3. What would be necessary for you to feel comfortable using a new AI technology for medical interpretation or document translation? *Probe on comparative studies, administration via human interpreters and translators, regulations, etc.*

**Caregiver Interview Guide**

1. To start, please tell me about what it has been like to access medical care for your child.

***Translation Current Practices***

*Thank you for sharing an overview of your experience with medical professionals and the various ways in which you communicate with them. We would now like to ask you some targeted questions about* ***translation*** *services (i.e., services used for* ***written*** *communication with your healthcare provider). Examples include written medication or discharge instructions, consent forms, electronic health record / chart notes, patient portal messages, etc.*

1. Please describe the most common ways that you communicate through written materials with your child’s healthcare providers.
   1. What resources or methods do you find most helpful?
   2. What are some challenges you face with written communication?
2. Please describe any situations where you were unable to understand or communicate through written documents. *Ask the interviewee to share specific examples*
   1. What did you do in this situation to try to overcome this communication barrier?
3. When you leave the doctor's office or hospital, do you have any challenges when you need to refer back to written material provided by your healthcare provider?

***Interpretation Current Practices***

*Now, we would now like to ask you some more targeted questions about* ***interpretation*** *services (i.e., services used to* ***verbally*** *communicate with providers in a language other than English). Examples include an appointment or encounter in the hospital or clinic, telehealth visit, or phone call.*

1. Please describe the most common ways you verbally communicate with healthcare providers in an office and/or hospital who do not speak your language.
   1. What resources or methods do you find most helpful?
   2. What are some challenges you face when communicating verbally?
2. Please describe any situations where you were unable to understand or communicate verbally with your child’s healthcare provider. *Ask the interviewee to share specific examples*
   1. What did you do in this situation to try to overcome this communication barrier?

*Now we will ask you some questions about artificial intelligence language technologies. Artificial intelligence is defined as computer systems that perform tasks that normally require human intelligence and can be used in a very wide range of settings.*

***Current Use & Opinions of Language Technologies***

1. What technologies, other than a human translator, have you used to communicate with your child’s healthcare providers?
   1. What went well and what did not go well?
   2. When might it be important not to use these services?
2. [If not already mentioned] What do you think about using technologies (other than humans) for translation and interpretation in a healthcare setting? *Probe on ChatGPT*
3. What would be necessary for you to feel comfortable using a new technology for medical interpretation or document translation? *Probe on comparative studies, administration via human interpreters and translators, etc.*
