# Supplementary material for: Caregiver and Pediatric Clinician Perspectives on Artificial Intelligence for Language Services
Source: Acad Pediatr. Author manuscript; Available in PMC 2026 Jul 7. (PMC13341081; doi:10.1016/j.acap.2025.102887)
Supplement: 2 [file NIHMS2170835-supplement-2.pdf]

APPENDIX 2. SURVEYS

CLINICIAN SURVEY

1. What is your role/specialty?
2. What percent of your time is spent in clinical practice?
3. How long have you been in practice?
4. On average, what percent of your patients primarily speak a language other than English?
5. Please share your racial and ethnic background. Please select ALL that apply. Under each category, you can describe more about where you trace your heritage and ancestry (eg, under Asian or Pacific Islander, you could share Nepali).
   a. Asian (describe if desired)
   b. Black, African, or African American (describe if desired)
   c. Hispanic, Latino, Latina, Latine, or Latinx (describe if desired)
   d. Indigenous, American Indian, or Alaskan Native (describe if desired)
   e. Middle Eastern/North African (describe if desired)
   f. Pacific Islander or Native Hawaiian (describe if desired)
   g. White or Caucasian (describe if desired)
   h. Some other race or ethnicity (please describe)
   i. Prefer not to say
6. Please share your gender identity. (Select all that apply)*Cisgender: I identify with the gender I was assigned at birth.Transgender: I identify with a different gender than I was assigned at birth.*
   a. Cisgender male/man
   b. Cisgender female/woman
   c. Transgender male/man
   d. Transgender female/woman
   e. Nonbinary
   f. Genderqueer
   g. Prefer to self-describe (write in)
   h. Prefer not to say

CAREGIVER SURVEY

1. What is your age?
2. What is your race and/or ethnicity?
3. What is your gender?
4. What is your preferred language(s)?
